# Magnetic graphs for cavity quantum electrodynamics

**DOI:** 10.1126/sciadv.aee5566

**Published:** 2026-09-25

**Authors:** Sunkyu Yu, Xianji Piao, Namkyoo Park

**Affiliations:** ^1^ Intelligent Wave Systems Laboratory, Department of Electrical and Computer Engineering, Seoul National University, Seoul 08826, Korea.; ^2^ Wave Engineering Laboratory, School of Electrical and Computer Engineering, University of Seoul, Seoul 02504, Korea.; ^3^ Photonic Systems Laboratory, Department of Electrical and Computer Engineering, Seoul National University, Seoul 08826, Korea.

## Abstract

Strengthening light-matter coupling has become a central challenge in cavity quantum electrodynamics (QED), enabling ultrafast gate operations, qubit protection, and deterministic nonlinear optics. As the coupling increases, even the simplest configuration—an atom interacting with a quantized field—requires careful treatment, as exemplified by ongoing debates on quantum Rabi models (QRMs). Here we propose a magnetic graph model for single-atom cavity QED that offers a unified description of quantum dynamics from the weak- to the ultrastrong-coupling regime via graph connectivity. We demonstrate that the generalized QRM maps onto a complex bipartite graph of identical sites under Floquet boundary conditions. This framework captures the crossover from weak to deep-strong coupling via a single metric—the cost of disconnecting a nonmagnetic subgraph. We examine the mechanism underlying this connectivity transition, establishing the pivotal role of phase frustration. Scalable to many-body systems, this approach bridges graph theory and cavity QED, revealing complex-graph dynamics even in the simplest setting.

## INTRODUCTION

Single-atom light-matter interaction is a cornerstone of cavity quantum electrodynamics (QED). However, its theoretical framework—the quantum Rabi model (QRM)—remains under active development (*1*–*7*), particularly regarding its validity in the ultrastrong coupling (USC; 0.1 ≲ η ≲ 1) and deep-strong coupling (DSC; η ≳ 1) regimes (*8*, *9*), where η = κ/ω_0_ is the dimensionless coupling strength, with κ the coupling and ω_0_ the cavity frequency. The intricacies of handling the QRM are intertwined with the unprecedented dynamics observed in the USC and DSC regimes: the Schrödinger’s cat ground states (*10*), nonlinear optics with virtual photons (*11*), photon excitation using mechanical engineering (*3*), and dressed-picture dissipation (*12*).

The traditional η-based classification of the USC and DSC regimes is tied to the validity of specific approximations (*13*). In the dipole-gauge formulation, the USC regime is marked by the substantial influence of counter-rotating terms, which can be neglected under the rotating-wave approximation (RWA) for weak and strong coupling regimes. By contrast, the DSC dynamics are well captured by an adiabatic displaced-oscillator picture, while the RWA collapses completely.

Recent insights grounded in the gauge principle have highlighted the need for an alternative framework to understand the USC and DSC regimes (*1*, *5*–*7*, *14*–*16*). The gauge-invariant, Coulomb-gauge formulation of the QRM (*1*)—obtained by sequentially applying truncation and minimal coupling replacement—leads to the appearance of field operators of all orders, obscuring the conventional USC-DSC classification derived from earlier Coulomb- or dipole-gauge formulations. These higher-order interactions, which mediate long-range hopping between states, invalidate a simple description of the RWA developed under examining at most quadratic field operators. Simultaneously, the presence of long-range hopping inspires a graph-based perspective by analogy with shortcuts in complex networks (*17*).

Here, we show that a semi-infinite, weighted magnetic graph having topological features is hidden within the simplest quantum system. Starting from the generalized QRM, we develop its magnetic graph interpretation, in which the magnetism in our analysis is not an externally applied field but a synthetic property arising from gauge-invariant phases inherent to the QRM. This interpretation reveals that the USC and DSC regimes can be classified by a graph connectivity metric—the cost of isolating a large magnetically trivial subgraph. We analyze how topological features and connectivity bottlenecks contribute to this cost, demonstrating the critical impact of phase frustration in quantum state localization. Our proposal provides metrics that not only classify cavity QED via quantum state connectivity but also probe topological features in hybrid quantum systems.

## RESULTS

### Gauge-invariant QRM

**Fig. 1.**
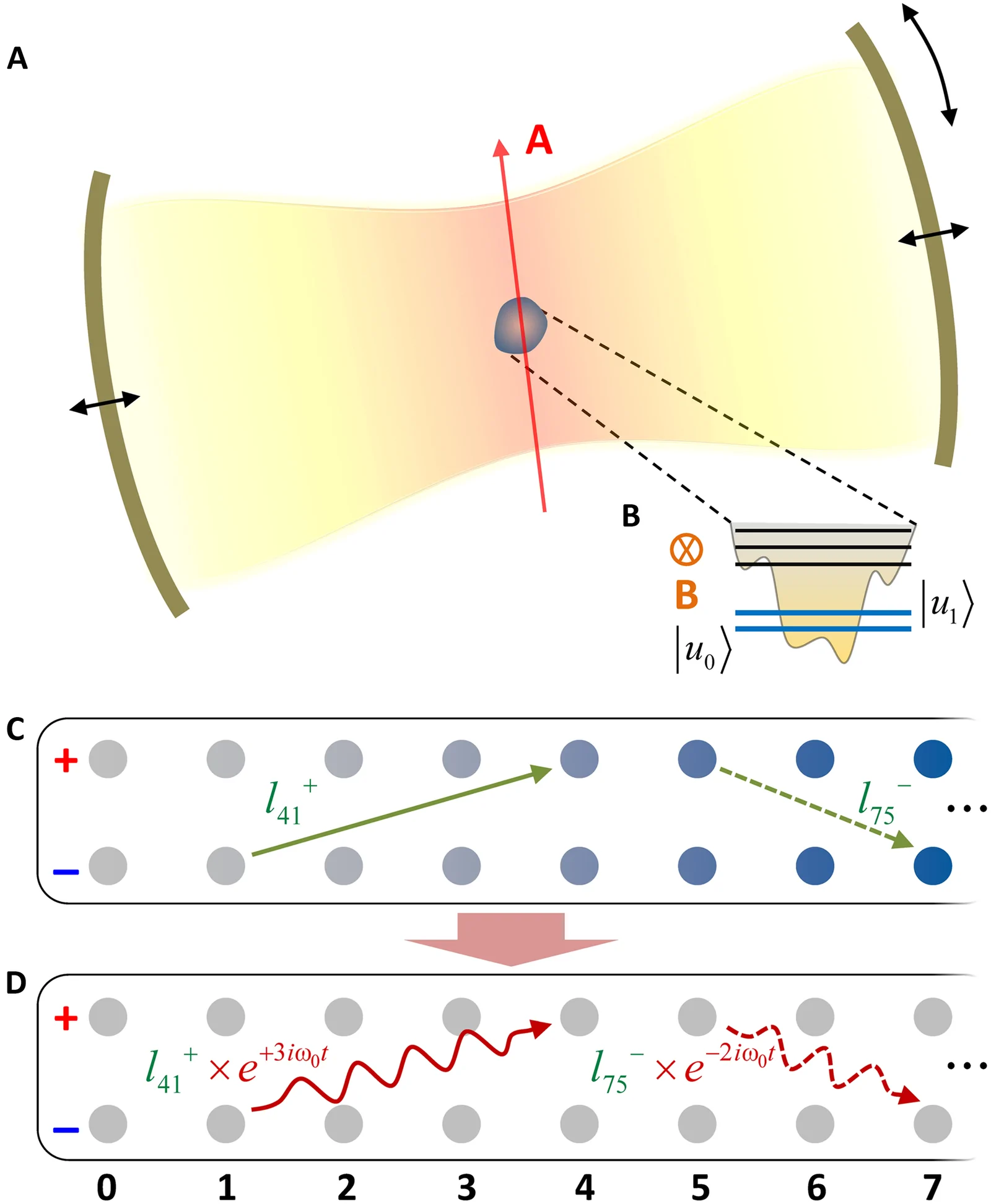
Magnetic graph model for single-atom cavity QED. (**A** and **B**) Schematics for the generalized QRM (A) and the eigenstates of an arbitrary atom (B). Black arrows in (A) indicate cavity modulations. (**C**) Graph model of Eq. 4, with linearly varying self-loops indicated by the graded color of the nodes (circles). (**D**) The FR graph obtained via a local gauge transformation of the graph in (C).

To develop a graph model for cavity QED, we start with a two-level atom coupled to a quantized cavity field (Fig. 1A). We use the gauge-invariant QRM, which provides a consistent description of time-dependent systems, particularly in the USC and DSC regimes (*1*). We extend the Hamiltonian *H*_C_ in the earlier work (*1*) to encompass a vector gauge field **A** = **A**_0_(*a*^†^ + *a*) (Fig. 1A) and generalized dipole moments that accommodate atoms with broken parity or time-reversal symmetry (Fig. 1B).

Consider an atom described by a bare Hamiltonian *H*_0_ = *h*_0_*I* + **h**·**σ** in the two-level subspace spanned by an orthonormal basis {|*u*_0_⟩, |*u*_1_⟩}, where *h*_0_ and **h** are a real-valued scalar and vector, respectively, **σ** = **i**σ*_x_* + **j**σ*_y_* + **k**σ*_z_* denotes the Pauli vector, and *I* is the identity matrix. The basis dependence is also encoded in the permanent dipole moments **d**_±_ = (**d**_11_ ± **d**_00_)/2, and the transition dipole moment **d** = **d**_r_ + *i***d**_i_ with **d**_r_ = Re[**d**_10_] and **d**_i_ = Im[**d**_10_], where **d***_rs_* = *q*⟨*u_r_*|**x**|*u_s_*⟩ (Materials and Methods, section Position operator and dipole moments). By applying the minimal coupling replacement (*1*), the QRM Hamiltonian becomes (Materials and Methods, section Generalized QRM)

$$H_{C}=\hbar \omega_{0}a^{†}a+h_{0}I+\left[h_{∥}+h_{+}D\left(+2i\eta \right)+h_{-}D\left(-2\mathrm{i\eta}\right)\right]\cdot \sigma$$ (1)

where η = |**g**|/ℏ is the dimensionless coupling strength in the Coulomb gauge obtained with **g** = (**A**_0_·**d**_r_)**i** – (**A**_0_·**d**_i_)**j** + (**A**_0_·**d**_−_)**k**, *D*(α) denotes the α-displacement operator, **h**_∥_ and **h**_⊥_ are the components of **h** parallel and perpendicular to **g**, respectively, and **h**_+_ and **h**_−_ are defined as

$$h_{\pm }=\frac{1}{2}\left(h_{⊥}\mp ih\times \frac{g}{|g|}\right)$$ (2)

Notably, **ĥ**_±_ = **h**_±_/|**h**_⊥_| and **ĥ**_∥_ = **h**_∥_/|**h**_∥_| constitute an orthogonal basis, leading to the spin basis states |φ_±_⟩ that satisfy (**ĥ**_∥_·**σ**)|φ_±_⟩ = ±|φ_±_⟩, (**ĥ**_±_·**σ**)|φ_±_⟩ = *O*, and (**ĥ**_∓_·**σ**)|φ_±_⟩ = ±|φ_∓_⟩. By using the eigenbasis {|*u*_0_⟩, |*u*_1_⟩} (Fig. 1B), cavity QED with **h** = *h_z_***k** is governed solely by **g**, which determines η—the conventional parameter used to classify the USC and DSC regimes (*1*, *13*). While a nonzero **d**_−_ indicates broken parity symmetry, broken time-reversal symmetry prevents cancellation of **d**_i_. The vector **A**_0_(*t*) accounts for dynamical cavity modulations (Fig. 1A). Consequently, Eq. 1 captures a broad class of realistic scenarios.

### Floquet Rabi graph

Inspired by *D*(α), which connects distant number states, we develop a graph model for the generalized QRM by using the Fock-state lattice representation (*18*–*21*). For the Schrödinger equation *i*ℏ|ψ⟩ = *H*_C_|ψ⟩, we use the ansatz |ψ⟩ = ∑*_n_*(*c_n_*^+^|φ_+_⟩ + *c_n_*^−^|φ_−_⟩)|*n*⟩, where |*n*⟩ denotes the number state. Substituting this ansatz into the Schrödinger equation yields (Materials and Methods, section Fock-state lattice representation)

$$\begin{array}{c} i\hbar \partial_{t}{c_{n}}^{+}=\left(n\hbar \omega_{0}+h_{0}+|h_{∥}|\right){c_{n}}^{+}+|h_{⊥}|\sum_{m=0}^{\infty }{l_{\mathrm{nm}}}^{+}{c_{m}}^{-}\\ i\hbar \partial_{t}{c_{n}}^{-}=\left(n\hbar \omega_{0}+h_{0}-|h_{∥}|\right){c_{n}}^{-}+|h_{⊥}|\sum_{m=0}^{\infty }{l_{\mathrm{nm}}}^{-}{c_{m}}^{+} \end{array}$$ (3)

where *l_nm_*^±^ = ⟨*n*|*D*(±2*i*η)|*m*⟩ determines the hopping between the sites |φ_±_⟩|*n*⟩ and |φ_∓_⟩|*m*⟩ (Fig. 1C), evaluated as

$${l_{\mathrm{nm}}}^{\pm }=e^{-2\eta^{2}}\sqrt{\frac{\text{min}\left\{m,n\right\}!}{\text{max}\left\{m,n\right\}!}}{\left(\pm 2i\eta \right)}^{|n-m|}{L_{\text{min}\left\{m,n\right\}}}^{|n-m|}\left(4\eta^{2}\right)$$ (4)

in terms of the associated Laguerre polynomial *L_a_*^(*b*)^(*x*). This hopping exhibits the asymptotic behaviors of *l_nm_*^±^(η → 0) = δ*_mn_* and *l_nm_*^±^(η → ∞) = 0 (Materials and Methods, section Asymptotic behaviors of hopping).

Owing to weighted hopping *l_nm_*^±^ = (*l_mn_*^∓^)^*^ that spans all orders of long-range hopping without Bloch boundary conditions, the model of Eq. 3 must be interpreted as a complex magnetic graph (*22*–*25*) rather than as a lattice. The QRM forms a bipartite graph composed of two disjoint semi-infinite sets, represented by the state vectors Ψ_±_ = [*c*_1_^±^, *c*_2_^±^, …]^T^, while the self-loops determined by onsite energies vary linearly as *n*ℏω_0_ with additional perturbations of ±|**h**_∥_|.

In characterizing the connectivity of a magnetic graph, a standard approach is to use the magnetic Laplacian (*25*), whose spectrum provides a systematic measure of subgraph separability. When constructing the magnetic Laplacian, it is common to set self-loops to zero (*25*) to isolate internode connectivity and to mitigate potential numerical issues arising from strong degree heterogeneity under normalization, such as the overemphasis of hub nodes. To enforce this configuration, we apply a gauge transformation that flattens the linear variation of the self-loops, which leads to [Materials and Methods, section Transformation to Floquet Rabi (FR) graph]

$$i\hbar \frac{\partial }{\partial t}\left[\begin{array}{c} \Psi_{+}\\ \Psi_{-} \end{array}\right]=\left[\begin{array}{cc} +|h_{∥}|I & {K_{\pm }}^{\prime }\\ {K_{\mp }}^{\prime } & -|h_{∥}|I \end{array}\right]\left[\begin{array}{c} \Psi_{+}\\ \Psi_{-} \end{array}\right]≜\Omega^{\prime }\left[\begin{array}{c} \Psi_{+}\\ \Psi_{-} \end{array}\right]$$ (5)

where [*K*_±_′]*_mn_* = exp[*i*(*m* – *n*)ω_0_*t*][*K*_±_]*_mn_* denotes the element of the transformed hopping matrix with [*K*_±_]*_mn_* = |**h**_⊥_|*l_mn_*^±^ (Fig. 1D). Because |*l_mn_*^±^| → 0 for sufficiently large |*m* – *n*| (Materials and Methods, sections Asymptotic behaviors of hopping), we can introduce a coupling strength–dependent maximum hopping order Δ*N*_η_ such that *l_mn_*^±^ ≈ 0 for |*m* – *n*| > Δ*N*_η_. This condition implies a finite temporal periodicity of 2π/[ω_0_Δ*N*_η_], yielding a Floquet graph composed of identical nodes except for the perturbations ±|**h**_∥_| on the disjoint states Ψ_±_. We refer to the resulting single-atom QED graph as the FR graph, denoted by *G*_FR_ = (*V*_FR_, *E*_FR_), where *V*_FR_ represents the set of quantum states |φ_±_⟩|*n*⟩ and *E*_FR_ denotes the set of edges characterized by *K*_±_′. Truncating the bosonic Hilbert space at *n* = *N*_max_ renders the FR graph finite.

**Fig. 2.**
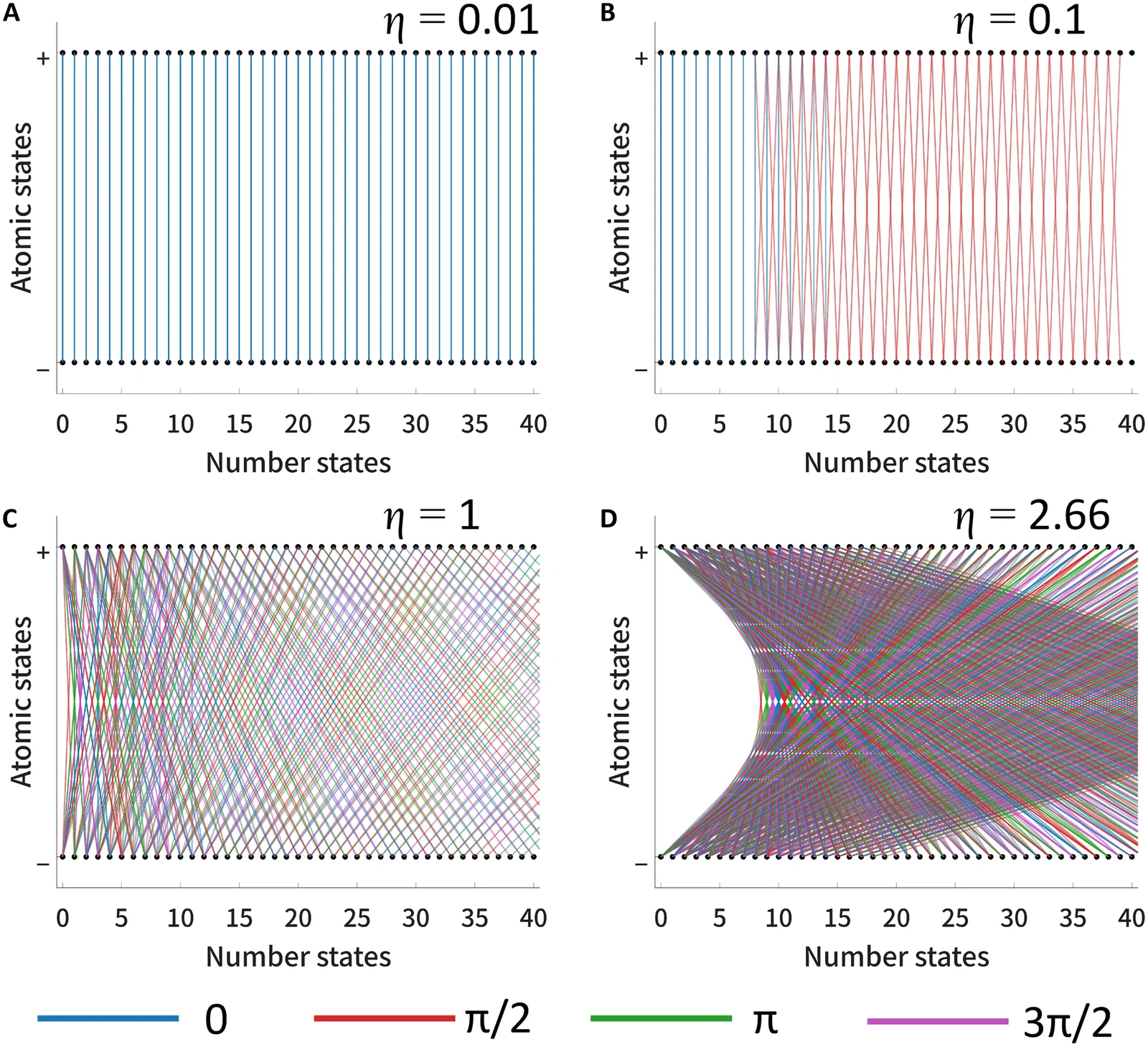
Graph connectivity for different values of η at *t* = 0. (**A** to **D**) Edge visualization for varying η. To ensure clarity, only edges with weights exceeding 50% of the global maximum are displayed for different values of η: η = 0.01 (A), η = 0.1 (B), η = 1 (C), and η = 2.66 (D). The strength of each weight is indicated by transparency. To suppress errors arising from finite-dimensional truncation of the Fock space, we use a large cutoff for the maximum number state: |*n* = *N*_max_ = 200⟩, which ensures negligible boundary effects over the range η ≤ 2.66 according to the following discussion of the magnetic Laplacian (note S1).

Figure 2 shows the FR graph connectivity depending on η. According to Eq. 4, the graph edges are classified by their hopping phases of γπ/2 (γ = 0, 1, 2, and 3), determined by the hopping order with the sign of the associated Laguerre polynomial. Although the nearest-neighbor (NN) edges are dominant in the weak-coupling regime (Fig. 2A), the effect of higher-order hopping increases with enhanced coupling (Fig. 2B), eventually comprising highly complicated mixing of complex-valued weights over the entire graph (Fig. 2, C and D).

### Magnetic Laplacian

To examine the connectivity of the FR graph—particularly in the USC and DSC regimes—we use the normalized magnetic Laplacian (*25*), while setting |**h**_∥_| = 0. For the magnetic graph operator Ω′ in Eq. 5, its Laplacian is *L*′ = *I* – *D*′^–1/2^Ω′*D*′^–1/2^, where *D*′ is the diagonal degree matrix constructed from the elementwise absolute value matrix |Ω′|. Although *L*′ = *L*′(*t*) has a Floquet form owing to *K*_±_′, it is readily proved that the eigenspectrum of *L*′ remains invariant under this temporal variation (Materials and Methods, section Magnetic Laplacian analysis for FR graph), indicating that the connectivity of the FR graph is preserved. Therefore, we investigate the graph connectivity using the static magnetic Laplacian *L* = *I* – *D*^–1/2^Ω*D*^–1/2^ with

$$\Omega =\left[\begin{array}{cc} 0 & K_{\pm }\\ K_{\mp } & 0 \end{array}\right]$$ (6)

where *D* is the diagonal degree matrix of |Ω|.

**Fig. 3.**
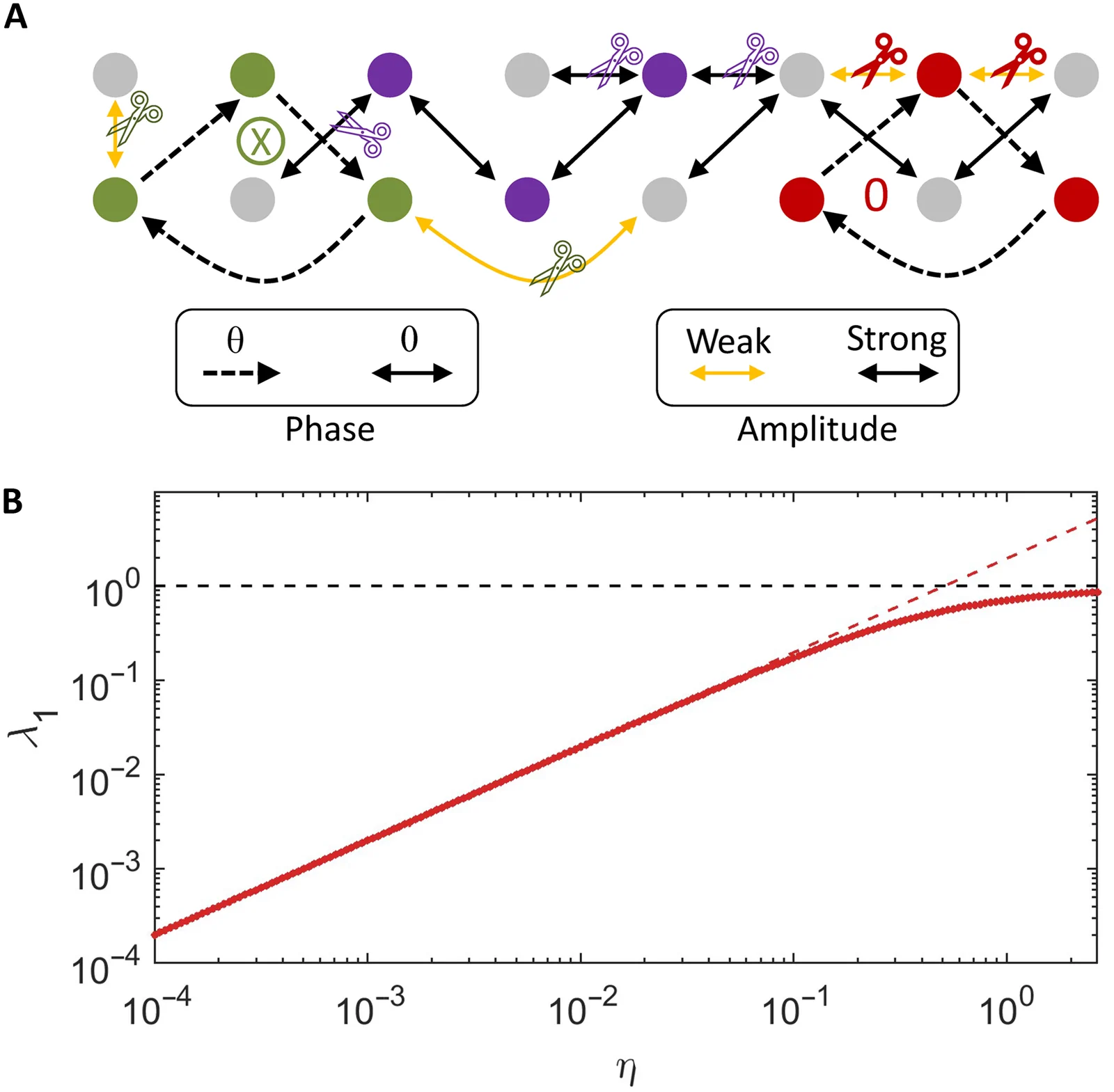
Stronger coupling indicates a higher cost for separating a large nonmagnetic subgraph. (**A**) Schematic of the generalized Cheeger’s inequality. Sets of green, purple, and red circles denote subgraphs. Scissors mark cuts—setting the edge weight to zero—used to separate subgraphs. Directional dashed arrows and double-headed solid arrows indicate hopping with nonzero and zero phase evolution, respectively. Black and orange arrows denote strong and weak hopping strengths, respectively. (**B**) λ_1_ as a function of η. The red and black dashed lines denote the weak-coupling asymptote and the upper bound on λ_1_, respectively.

Using the Laplacian, we solve the eigenvalue problem *Lv_m_ =* λ*_m_v_m_* for positive integers *m* and λ*_m_* ≤ λ_*m*+1_. According to the generalized Cheeger’s inequality (*25*), the critical metric for characterizing graph connectivity is the first eigenvalue 0 ≤ λ_1_ ≤ 1 (Materials and Methods, section Bounds on λ_1_), which provides a lower bound on the cost of separating a large phase-coherent (or magnetic flux–free) subgraph *G*_S_ ⊆ *G*_FR_. To illustrate this analysis, consider the example graph in Fig. 3A, having three subgraphs: green, purple, and red ones. In terms of phases and strengths of edge weights, the red subgraph corresponds to a phase-coherent—that is, the absence of a magnetic flux—region (“0”) with a low separation cost due to the weight bottleneck (orange arrows). By contrast, the green subgraph exhibits a nonzero magnetic flux—referred to as phase frustration in graph theory (*25*)—that must be avoided for coherence, while the purple one is strongly connected to the remainder of the graph and, therefore, enforces a high separation cost. Therefore, λ_1_ represents the minimum cost of separating the red subgraph from the graph.

Figure 3B shows the variation of λ_1_ with η over the range of η ≤ 2.66, for which the Laplacian eigenstate *v*_1_ has negligible weight near the boundary |*n* = *N*_max_ = 200⟩ (note S1). Over the weak- to strong-coupling range (η < 10^−1^), the plot simply follows the linear law λ_1_ = 2η. Within the interval of 0 ≤ λ_1_ ≤ 1, the regimes beyond the USC are characterized by substantial deviations from the linearity, whereas the DSC regime is identified by the saturation of λ_1_ at unity. Consequently, our FR graph enables a λ_1_-based classification of single-atom cavity QED, which continuously quantifies the cost of separating a nonmagnetic subgraph. We note that λ_1_ provides a single, continuous-valued metric derived directly from the connectivity of quantum states in the generalized QRM, avoiding the need to invoke regime-specific approximation conditions (*13*).

### Conductance and frustration

Although λ_1_ offers a single-parameter classification of single-atom cavity QED, an increase in λ_1_ with stronger coupling can originate from two distinct mechanisms: (i) a high separation cost due to the alleviation of edge-weight bottlenecks and (ii) magnetic-flux–induced phase frustration. Their impacts on quantum states, specifically in terms of transport over the FR graph, are opposite: Mitigating bottlenecks enhances transport—that is, higher conductance (*26*)—whereas the frustration with phase incoherence, leads to localization.

**Fig. 4.**
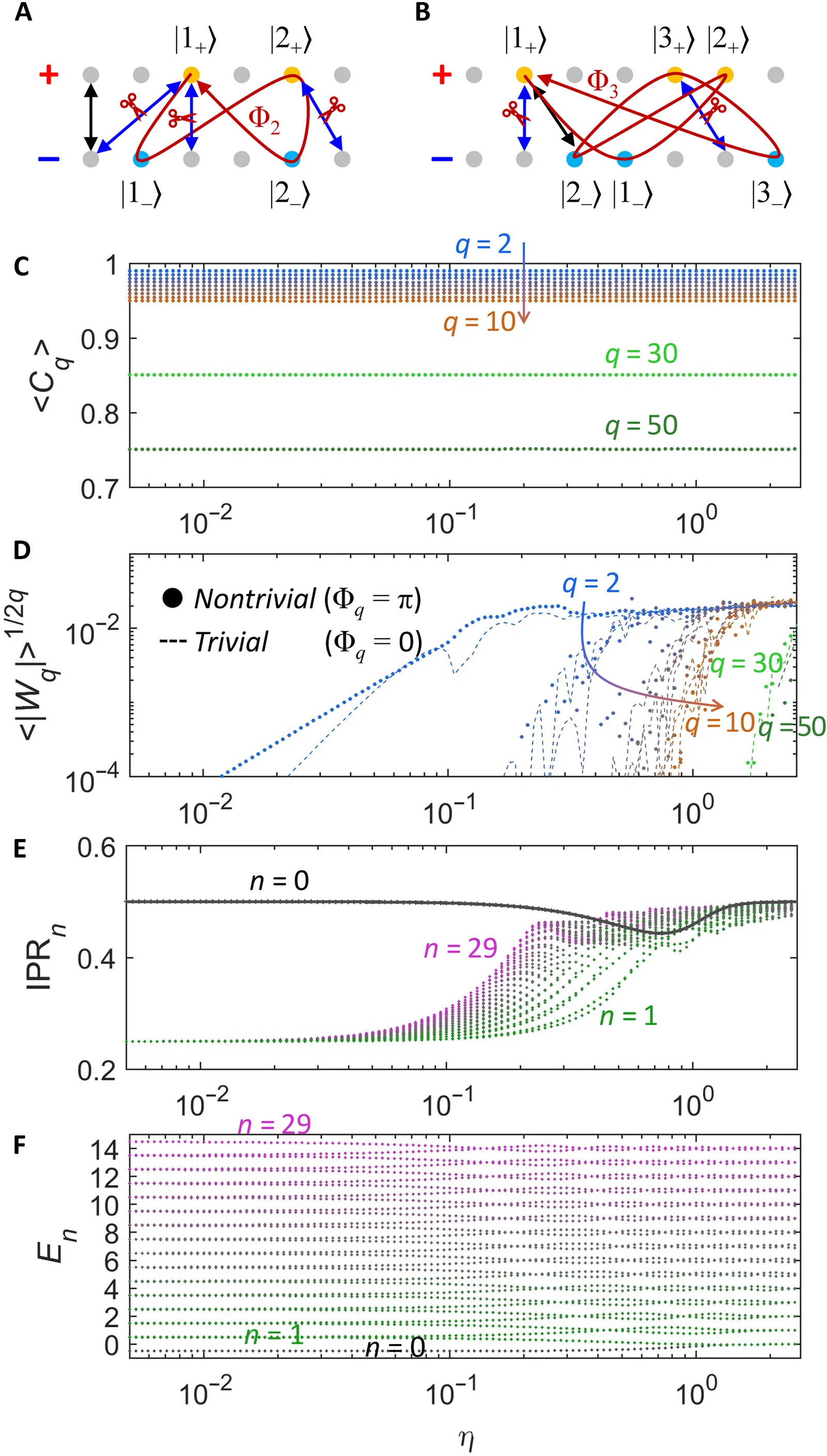
Phase frustration governs the USC. (**A** and **B**) Schematics of subgraphs (colored nodes) illustrating closed loops for *q* = 2 (A) and *q* = 3 (B). Black and blue arrows indicate example edges, while the blue ones must be cut to separate the subgraphs. Red curved arrows denote closed loops. (**C** and **D**) Graph measures: ⟨*C_q_*⟩ (C) and |*W_q_*|^1/(2*q*)^ (D), as functions of η, for different *q* values. Both measures are computed over 2 × 10^4^ random subgraphs, while |*W_q_*|^1/(2*q*)^ is evaluated separately for nontrivial (dots) and trivial (dashed lines) phases. (**E** and **F**) Eigenstate analysis: IPR (E) and *E*_n_ (F) as functions of η. The first 30 eigenstates are plotted.

To identify the origin of the variation in λ_1_, we disentangle the two contributions by randomly constructing subgraphs and examining their closed loops. Owing to the bipartite structure of the FR graph, each closed loop contains an equal number of |φ_+_⟩|*n*⟩ and |φ_−_⟩|*n*⟩ site vertices (Fig. 4, A and B). Accordingly, we form a subgraph, *G*_S_*^q^* = (*V*_S_*^q^*, *E*_S_*^q^*), by randomly selecting *q* pairs across the bipartition, as *V*_S_*^q^* = {(|*r*_+_⟩, |*s*_−_⟩)|*r*, *s* = 1, 2,…, *q*}, where |*r*_±_⟩ ≔ |φ_±_⟩|*n*(*r*_±_)⟩, and *n*(*r*) ∼ Unif{0, 1, 2, …, *N*_max_} denotes uniform sampling over the number states. For *q* > 2, a closed loop can be defined as *e*(1_−_, 1_+_) → *e*(2_+_, 1_−_) → *e*(2_−_, 2_+_) → … → *e*(1_+_, *q*_−_,), where *e*(*r*_σ_, *s*_τ_) ∈ *E*_S_*^q^* is the directed edge from |*s*_τ_⟩ to |*r*_σ_⟩ with complex weight *w*(*r*_σ_, *s*_τ_) for σ, τ = (±).

For each subgraph *G*_S_*^q^* composed of 2*q* nodes and one of its closed loops Γ*_q_*, we evaluate the origin of the λ_1_ variation using the conductance and the loop weight

$$C_{q}=\frac{\sum_{r_{\sigma }\in {\left({V_{S}}^{q}\right)}^{C},s_{\tau }\in {V_{S}}^{q}}|w\left(r_{\sigma },s_{\tau }\right)|}{\sum_{r_{\sigma }\in {V_{S}}^{q},s_{\tau }\in V_{\text{FR}}}|w\left(r_{\sigma },s_{\tau }\right)|},W_{q}=\prod_{e\left(r_{\sigma },s_{\tau }\right)\in \Gamma_{q}}w\left(r_{\sigma },s_{\tau }\right)$$ (7)

where their *q*-dependencies characterize the degree of globality of these two measures. The conductance *C_q_* measures the total strength of edges connecting *G*_S_*^q^* to the rest of the graph, normalized by the volume of *G*_S_*^q^*—the sum of node degrees. This quantity characterizes the separation cost of *G*_S_*^q^* by probing the presence of bottlenecks (*26*).

The magnetic signature of the FR graph is quantified by *W_q_* = |*W_q_*|exp.(*i*Φ*_q_*). Its phase Φ*_q_* is a topological invariant quantized to 0 (trivial) or π (nontrivial) modulo 2π, which characterizes the parity conversion of the number states along the loop (Materials and Methods, section Topological magnetic fluxes). Consequently, a large fraction of subgraphs having nontrivial phase Φ*_q_* = π leads to destructive interference and the resulting phase frustration, thereby contributing to increasing λ_1_. Because the FR graph is weighted, this fraction is weighted by |*W_q_*|.

Figure 4C shows the ensemble-averaged conductance ⟨*C_q_*⟩ over random subgraphs. The result demonstrates that conductance does not contribute to the λ_1_ variation, as ⟨*C_q_*⟩ remains nearly constant with respect to η; that is, the fraction of bottlenecks reflecting the graph structure is independent of η. Moreover, the approximately linear dependence of ⟨*C_q_*⟩ on the subgraph size 2*q* indicates that the FR graph is quasi–one dimensional, reflecting the dimensional distinction between |φ_±_⟩ and |*n*⟩. Consequently, the coupling-induced change in graph connectivity originates solely from phase frustration. To verify this prediction, we evaluate *W_q_*. In the weak and strong coupling regimes, the graph connectivity is dominated by NN hopping, hindering the closed-loop formation. As η increases, the system progressively enables higher *q*-loop formation, reaching >80% for *q* = 2 and > 40% for *q* = 10 as it approaches the DSC regime (note S2).

Using the loop formation, we compute the ensemble-averaged |*W_q_*|^1/(2*q*)^—the averaged connection strength (Materials and Methods, section Connection strengths)—separately for nontrivial (Φ*_q_* = π) and trivial (Φ*_q_* = 0) loops (Fig. 4D). In parallel, we evaluate the eigenstates of *H*_C_|ψ*_n_*⟩ = *E_n_*|ψ*_n_*⟩ via two measures: the inverse participation ratio (IPR) ${\text{IPR}}_{n}=\sum_{v=1}^{2\left(N_{\text{max}}+1\right)}{|\langle v|\psi_{n}\rangle |}^{4}$, which quantifies the state localization (Fig. 4E) and the eigenvalues *E_n_* (Fig. 4F). The results demonstrate that phase frustration governs the coupling-induced state transition in cavity QED. As shown in Fig. 4D, approximately half of the formed loops are nontrivial. The destructive interference induced by these nontrivial loops leads to localization (Fig. 4E) and the consequent spectral degeneracies (Fig. 4F). While the weak- and strong-coupling regimes are dominated by *q* = 2 loops, the USC regime is characterized by the mixed emergence of higher-order loops (*q* > 2). We note that |*W_q_*|^1/(2*q*)^ saturates as the system approaches the DSC regime across different *q* values, indicating that phase frustration becomes uniform across the graph, leading to similar values of ⟨Φ*_q_*⟩ regardless of *q* or loop configuration.

## DISCUSSION

Although we apply the magnetic graph modeling to single-atom cavity QED, the approach can be readily extended to many-body systems, such as the Dicke and Rabi-Hubbard models. Using the Coulomb-gauge models developed under the gauge principle (*1*, *14*), the emergence of long-range hopping in the Fock-state lattice can be transformed into a large-scale magnetic graph with identical nodes under Floquet boundary conditions. This extension enables the analysis of large-scale quantum phenomena using subgraph separation and phase frustration. Therefore, our graph model can operate as a general framework for classifying cavity QED including many-body phenomena.

The graph-theoretic signatures of the generalized QRM, originating from the connectivity crossover encoded in λ_1_, can be mapped to experimentally accessible observables. While quantum state localization and spectral features can be probed via anomalous photon-number statistics in cavity QED (*10*) and microwave transmission spectroscopy in superconducting quantum circuits (*27*), respectively, the probability distribution over the FR graph can be experimentally reconstructed by quantum state tomography (*28*). In addition, because our graph model based on the generalized QRM (*1*) incorporates temporal cavity modulations via **A**_0_(*t*), driven and time-resolved measurements could reveal the dynamical consequences of varying graph connectivity. More broadly, such experimentally measured observables, together with our magnetic graph framework, provide a connection between the underlying quantum dynamics and graph-based learning models (*29*), potentially enabling scalable, data-driven simulations of quantum phenomena.

In conclusion, we investigated complex graph dynamics in the simplest quantum system. For the generalized QRM that incorporates vector gauge fields and cavity modulations, we constructed its magnetic graph representation. We quantified graph connectivity through λ_1_, which indicates the USC and DSC regimes. We analyzed the origin of λ_1_ in terms of separation cost and phase frustration, demonstrating the critical role of frustration. This approach provides a universal framework for interpreting atom-light interactions as a complex graph, enabling the characterization of quantum-state properties, such as robustness from localization on the graph. The application of graph-based methods, including spectral clustering and graph neural networks, would be promising future research.

## MATERIALS AND METHODS

### Position operator and dipole moments

Following the procedures in previous works (*1*, *14*), we sequentially apply truncation and minimal coupling replacement to the Schrödinger equation in the infinite-dimensional Hilbert space H. As a preliminary step, we prepare projection-based representation of the position operator and the dipole moments. Consider an arbitrary orthonormal basis {|*u_n_*⟩|*n* = 0, 1, …, ∞} of H. To express the position operator, we introduce the projection operator

$$P=\sum_{r\in N_{T}}|u_{r}\rangle \langle u_{r}|$$ (8)

which leads to **x** = *P***x***P* when *N*_T_ = {0, 1, …, ∞}, and thus, *P* = *I*. The position operator is represented as

$$x=\sum_{r,s\in N_{T}}\frac{d_{\mathrm{rs}}}{q}|u_{r}\rangle \langle u_{s}|$$ (9)

where **d***_rs_* = *q*⟨*u_r_*|**x**|*u_s_*⟩ corresponds to the vector dipole matrix element between the *r*-th and *s*-th basis vectors.

### Generalized QRM

To analyze a single-atom cavity QED, the infinite-dimensional Hilbert space H needs to be truncated to a two-level subspace $H_{T}$. This corresponds to introducing a truncated index set *N*_T_ = {0, 1} in Eqs. 8 and 9, indicating two states {|*u*_0_⟩, |*u*_1_⟩} that yield physics well separated from that of the remaining states (Fig. 1B). An arbitrary bare Hamiltonian *H*_0_ on $H_{T}$ then becomes *H*_0_ = *h*_0_*I* + **h**·**σ**. When |*u*_0_⟩ and |*u*_1_⟩ are spatially localized, the dipole moment vectors are also well defined in $H_{T}$: the averaged and differential permanent dipole moments **d**_±_ = (**d**_11_ ± **d**_00_) / 2 and the transition dipole moment **d** = **d**_r_ + *i***d**_i_ with **d**_r_ = Re[**d**_10_] and **d**_i_ = Im[**d**_10_].

According to minimal coupling replacement with a single-mode gauge field **A** = **A**_0_(*a*^†^ + *a*) under the dipole approximation, the gauge-invariant Hamiltonian in the Coulomb gauge is obtained as *H*_C_ = *UH*_0_*U*^†^ + ℏω_0_*a*^†^*a* (*1*), where ω_0_ denotes the frequency of light, and *U* = exp[(*i*/ℏ)*q***A**·**x**] is the unitary operator on $H_{T}$. Using the truncated position operator, **x** = (1/*q*)(**d**_+_*I* + **d**_r_σ*_x_* – **d**_i_σ*_y_* + **d**_−_σ*_z_*), we obtain *U* = exp[(*i*/ℏ)(*g*_0_*I* + **g**·**σ**)(*a*^†^ + *a*)], where *g*_0_ = **A**_0_·**d**_+_ and **g** = (**A**_0_·**d**_r_)**i** – (**A**_0_·**d**_i_)**j** + (**A**_0_·**d**_−_)**k**.

To obtain *H*_C_ = *UH*_0_*U*^†^ + ℏω_0_*a*^†^*a*, we use the BCH formula (*30*). When setting *X* = (*i*/ℏ)(*g*_0_*I* + **g**·**σ**)(*a*^†^ + *a*) for *U* = exp[(*i*/ℏ)(*g*_0_*I* + **g**·**σ**)(*a*^†^ + *a*)], and *Y* = *H*_0_ = *h*_0_*I* + **h**·**σ**, their commutation gives

$$\left[X,Y\right]=\frac{2}{\hbar }\left(a^{†}+a\right)\left(h\times g\right)\cdot \sigma$$ (10)

Through the recursive calculation, we obtain

$${\left[X,Y\right]}_{m}={\left(\frac{2}{\hbar }\right)}^{m}{\left(a^{†}+a\right)}^{m}{T_{g}}^{m}\left(h\right)\cdot \sigma$$ (11)

where [*X*,*Y*]*_m_* denotes a series of the commutation satisfying [*X*,*Y*]*_m_* = [*X*,[*X*,*Y*]_*m*–1_] with [*X*,*Y*]_1_ = [*X*,*Y*], and *T*_**g**_*^m^*(**h**) denotes a series of the cross product as *T*_**g**_*^m^*(**h**) = *T*_**g**_^*m*–1^(**h**) × **g** and *T*_**g**_^0^(**h**) = **h**. Therefore, the BCH formula for *e^X^Ye*^–*X*^ leads to the following gauge-invariant Hamiltonian

$$\begin{array}{c} H_{C}=\hbar \omega_{0}a^{†}a+h_{0}I+\sum_{m=0}^{\infty }\frac{1}{m!}{\left(\frac{2}{\hbar }\right)}^{m}{\left(a^{†}+a\right)}^{m}{T_{g}}^{m}\left(h\right)\cdot \sigma \\ ≜\hbar \omega_{0}a^{†}a+h_{0}I+\left[\text{exp}\left(2\frac{a^{†}+a}{\hbar }T_{g}\right)h\right]\cdot \sigma \end{array}$$ (12)

By applying the Rodrigues rotation formula (*31*), we obtain

$$\begin{array}{c} H_{C}=\hbar \omega_{0}a^{†}a+h_{0}I+\left[h_{∥}+h_{⊥}\text{cos}\left(\frac{2\left(a^{†}+a\right)}{\hbar }|g|\right)\right.\\ \left.+h\times \frac{g}{|g|}\text{sin}\left(\frac{2\left(a^{†}+a\right)}{\hbar }|g|\right)\right]\cdot \sigma \end{array}$$ (13)

where **h**_∥_ and **h**_⊥_ denote the parallel and perpendicular components of **h** with respect to **g**, respectively, as **h**_∥_ = (**h**·**g**)**g**/|**g**|^2^ and **h**_⊥_ = **h** – **h**_∥_.

Using the displacement operator (*32*), *D*(α) = exp(α*a*^†^ − α^*^a), the trigonometric operators in Eq. 13 can be expressed in terms of *D*, as cos[α(*a*^†^ + *a*)] = [*D*(+*i*α) + *D*(−*i*α)]/2 and sin[α(*a*^†^ + *a*)] = [*D*(+*i*α) – *D*(−*i*α)]/2*i*. By substituting these relations into Eq. 13, we obtain Eqs. 1 and 2.

Equation 1 is independent of the chosen basis {|*u*_0_⟩, |*u*_1_⟩}, allowing one to treat an arbitrary Hamiltonian *H*_0_ characterized by **h**, together with the generalized vector dipole moments, **d**_±_ and **d**. We note that **d**_−_ and **d**_i_ can be set to zero by choosing {|*u*_0_⟩, |*u*_1_⟩} to satisfy parity and time-reversal symmetries. In this case, the system is governed by the Hamiltonian vector **h** and the simplified vector **g** = (**A**_0_·**d**_r_)**I**, which depends solely on the vector field **A**_0_.

However, for consistency with experimental measurements, the eigenbasis {|*u*_0_⟩, |*u*_1_⟩} defined by *H*_0_|*u_n_*⟩ = *E_n_*|*u_n_*⟩ is conventionally used. Under the two-level truncation, we obtain *H*_0_ = [(*E*_1_ + *E*_0_)/2]*I* + [(*E*_1_ – *E*_0_)/2]σ*_z_*, leading to **h** = (*E*_10_/2)**k** for *E*_10_ = *E*_1_ – *E*_0_. In this representation, the vector **g** encodes the coupling between dipole moments and light.

### Fock-state lattice representation

When applying *H*_C_ in Eq. 1 to the ansatz |ψ⟩ = ∑*_n_*(*c_n_*^+^|φ_+_⟩ + *c_n_*^−^|φ_−_⟩)|*n*⟩, we obtain

$$\begin{array}{c} H_{C}|\psi \rangle =\sum_{m}\left(m\hbar \omega_{0}+h_{0}\right)\left({c_{m}}^{+}|\varphi_{+}\rangle +{c_{m}}^{-}|\varphi_{-}\rangle \right)|m\rangle \\ +|h_{∥}|\left({c_{m}}^{+}|\varphi_{+}\rangle -{c_{m}}^{-}|\varphi_{-}\rangle \right)|m\rangle \\ +|h_{⊥}|\left({c_{m}}^{-}|\varphi_{+}\rangle \right)D\left(+2i\eta \right)|m\rangle \\ +|h_{⊥}|\left({c_{m}}^{+}|\varphi_{-}\rangle \right)D\left(-2i\eta \right)|m\rangle \end{array}$$ (14)

using the relations: (**ĥ**_∥_·**σ**)|φ_±_⟩ = ±|φ_±_⟩, (**ĥ**_±_·**σ**)|φ_±_⟩ = *O*, and (**ĥ**_∓_·**σ**)|φ_±_⟩ = ±|φ_∓_⟩. By exerting ⟨φ_+_|⟨*n*| and ⟨φ_−_|⟨*n*| on the right side of Eq. 14, we obtain Eq. 3 with *l_nm_*^±^ = ⟨*n*|*D*(±2*i*η)|*m*⟩.

### Asymptotic behaviors of hopping

In analyzing the hopping coefficient, *l_nm_*^±^ in Eq. 4, we introduce the coefficients *s* ≔ min{*m*,*n*} and Δ ≔ |*m* – *n*| for simplicity, which lead to

$${l_{\mathrm{nm}}}^{\pm }=e^{-2\eta^{2}}\sqrt{\frac{s!}{\left(s+\Delta \right)!}}{\left(\pm 2i\eta \right)}^{\Delta }{L_{s}}^{\Delta }\left(4\eta^{2}\right)$$ (15)

The associated Laguerre polynomial has the closed form of

$${L_{s}}^{\Delta }\left(4\eta^{2}\right)=\sum_{k=0}^{s}{\left(-1\right)}^{k}\left(\begin{array}{c} s+\Delta \\ s-k \end{array}\right)\frac{{\left(4\eta^{2}\right)}^{k}}{k!}$$ (16)

When η → 0, we use exp(−2ℏη^2^) = 1 – 2ℏη^2^ + *O*(η^4^), achieving the following asymptotic behavior

$$\begin{array}{c} {l_{\mathrm{nm}}}^{\pm }=\frac{{\left(\pm 2i\eta \right)}^{\Delta }}{\Delta !}\sqrt{\frac{\left(s+\Delta \right)!}{s!}}\left[1-2\left(1+\frac{2s}{\Delta +1}\right)\eta^{2}\right]\\ +O\left(\eta^{4}\right) \end{array}$$ (17)

which gives *l_nn_*^±^ = 1 – 2(1 + 2n)η^2^ + *O*(η^4^). Therefore, in the weak coupling regime, the (±2*i*η)^Δ^ term in Eq. 17 indicates that the NN hopping between spin states *l_nn_*^±^ dominates the QRM dynamics.

When η → ∞, the higher-order terms in Eq. 16 dominate, as *L_s_*^Δ^(4η^2^) = (−1)*^s^*(4η^2^)*^s^*[1 – *s*(*s* + Δ)/(4η^2^)]/*s*! + *O*(η^2*s*–4^), which leads to

$$\begin{array}{c} {l_{\mathrm{nm}}}^{\pm }=\frac{{\left(\pm i\right)}^{\Delta }{\left(-1\right)}^{s}}{\sqrt{s!\left(s+\Delta \right)!}}{\left(2\eta \right)}^{m+n}e^{-2\eta^{2}}\\ \times \left[1-\frac{s\left(s+\Delta \right)}{4\eta^{2}}+O\left(\eta^{-4}\right)\right] \end{array}$$ (18)

Therefore, in the DSC regime with η ≫ 1, *l_nm_*^±^ decays as the Gaussian, exp(−2η^2^), multiplied by the polynomial factor η^*m*+*n*^. For a given large value of η, the increase of *s* = min{*m*,*n*} or Δ = |*m* – *n*| leads to |*l_nm_*^±^| → 0.

### Transformation to FR graph

For Ψ_±_ = [*c*_1_^±^, *c*_2_^±^, …]^T^, Eq. 3 can be rewritten as

$$i\hbar \frac{\partial }{\partial t}\left[\begin{array}{c} \Psi_{+}\\ \Psi_{-} \end{array}\right]=\left[\begin{array}{cc} B+|h_{∥}|I & K_{\pm }\\ K_{\mp } & B-|h_{∥}|I \end{array}\right]\left[\begin{array}{c} \Psi_{+}\\ \Psi_{-} \end{array}\right]$$ (19)

where *K*_±_ denotes the hopping matrices with [*K*_±_]*_mn_* = |**h**_⊥_|*l_mn_*^±^, and *B* is the diagonal matrix given by [*B*]*_nn_* = *n*ℏω_0_ + *h*_0_. To apply the analytical tool for the connectivity of magnetic graphs (*22*–*25*), we need to eliminate *B* ± |**h**_∥_|*I* in Eq. 19. The term |**h**_∥_| can be treated as a perturbation because it vanishes when the atomic potential satisfies parity symmetry with |**d**_−_| = 0. To remove the remaining terms, we apply the following gauge transformation analogous to the standard treatment of Wannier-Stark ladders and Bloch oscillations (*33*, *34*)

$$\begin{array}{c} i\hbar \frac{\partial }{\partial t}\left[\begin{array}{c} {\Psi_{+}}^{\prime }\\ {\Psi_{-}}^{\prime } \end{array}\right]=U\left[\begin{array}{cc} B+|h_{∥}|I & K_{\pm }\\ K_{\mp } & B-|h_{∥}|I \end{array}\right]U^{†}\left[\begin{array}{c} {\Psi_{+}}^{\prime }\\ {\Psi_{-}}^{\prime } \end{array}\right]\\ +i\hbar \left(\frac{\partial U}{\partial t}\right)U^{†}\left[\begin{array}{c} {\Psi_{+}}^{\prime }\\ {\Psi_{-}}^{\prime } \end{array}\right] \end{array}$$ (20)

where the transformation is defined by

$$U=\left[\begin{array}{cc} V & O\\ O & V \end{array}\right],V=\text{exp}\left[\frac{i}{\hbar }\sum_{n}\left(n\hbar \omega_{0}+h_{0}\right)|n\rangle \langle n|\right]$$ (21)

We then obtain

$$i\hbar \frac{\partial }{\partial t}\left[\begin{array}{c} {\Psi_{+}}^{\prime }\\ {\Psi_{-}}^{\prime } \end{array}\right]=\left[\begin{array}{cc} +|h_{∥}|I & {\mathrm{UK}}_{\pm }U^{†}\\ {\mathrm{UK}}_{\mp }U^{†} & -|h_{∥}|I \end{array}\right]\left[\begin{array}{c} {\Psi_{+}}^{\prime }\\ {\Psi_{-}}^{\prime } \end{array}\right]$$ (22)

which becomes Eq. 5 upon redefining Ψ_±_ ≔ Ψ_±_′.

### Magnetic Laplacian analysis for FR graph

Consider the Floquet magnetic Laplacian of the FR graph: *L*′(*t*) = *I* – *D*′^–1/2^Ω′*D*′^–1/2^, where

$$\Omega^{\prime }=\left[\begin{array}{cc} 0 & {K_{\pm }}^{\prime }\\ {K_{\mp }}^{\prime } & 0 \end{array}\right]=V\left[\begin{array}{cc} 0 & K_{\pm }\\ K_{\mp } & 0 \end{array}\right]V^{†}$$ (23)

for the diagonal matrix *V* in Eq. 21. Therefore, we can obtain *L*′(*t*) = *I* – *VD*′^–1/2^Ω′*D*′^–1/2^*V*^†^ for diagonal matrices *V* and *D*′*.* We also note that |Ω′| = |Ω| because of the relationship [*K*_±_′]*_mn_* = exp[*i*(*m* – *n*)ω_0_*t*][*K*_±_]*_mn_*, which leads to *D* = *D*′. Therefore, the relationship between the Floquet magnetic Laplacian and the static magnetic Laplacian becomes

$$L=V^{†}L^{\prime }\left(t\right)V$$ (24)

Because a unitary transformation preserves the eigenspectrum, we can analyze the connectivity of the FR graph using *L*.

### Bounds on λ_1_

Because *L* is positive semidefinite, its smallest eigenvalue satisfies λ_1_ ≥ 0. We also note that the sum of the eigenvalues equals Tr[*L*]. Using the cyclicity of the trace, we obtain

$$\text{Tr}\left[L\right]=\text{Tr}\left[I\right]-\text{Tr}\left[\Omega D^{-1}\right]=N$$ (25)

because Tr[Ω*D*^−1^] = 0 with [Ω]*_nn_* = 0 and the diagonal matrix *D*. The average of the eigenvalues is then 1. Because λ_1_ is no greater than this average for the positive semidefinite *L*, we obtain 0 ≤ λ_1_ ≤ 1.

### Topological magnetic fluxes

Consider a closed loop, *e*(1_−_, 1_+_) → *e*(2_+_, 1_−_) → *e*(2_−_, 2_+_) → … → *e*(1_+_, *q*_−_,), with the corresponding complex edge weights *w*(*r*_σ_, *s*_τ_) for σ, τ = (±). According to Eqs. 4 and 6, the weight *w*(*r*_σ_, *s*_τ_) can have a hopping phase γπ/2 (γ = 0, 1, 2, and 3), where even and odd values of γ correspond to even and odd values of |*n*(*r*_σ_) – *n*(*s*_τ_)|, respectively. Therefore, the number of edges with odd γ can be interpreted as counting the number of parity conversions along the sequence {*n*(1_+_), *n*(1_−_), *n*(2_+_), *n*(2_−_), …, *n*(*q*_+_), *n*(*q*_−_), *n*(1_+_)}. Because the sequence starts and ends with *n*(1_+_), which has the same parity, the number of parity conversions must be even. Therefore, the accumulated phase Φ*_q_* is quantized to 0 (trivial) or π (nontrivial). The quantized value reflects the topological nature of the weighted loop defined by the sequence {*n*(1_+_), *n*(1_−_), *n*(2_+_), *n*(2_−_), …, *n*(*q*_+_), *n*(*q*_−_), *n*(1_+_)}.

### Connection strengths

According to Eq. 7, *W_q_* is defined as the multiplication of 2*q* weights *w*(*r*_σ_, *s*_τ_). When any of these weights is zero, *W_q_* becomes zero, indicating a disconnected loop. Otherwise, |*W_q_|*^1/(2*q*)^ quantifies the average connection strength per edge along the loop. This quantity allows for comparing loops with different values of *q*, including disconnected ones.

## Supplementary Materials

### The PDF file includes:

Supplementary Notes S1 and S2 Figs. S1 and S2 Legend for code S1

### Other Supplementary Material for this manuscript includes the following:

Code S1
