# Supplementary material for: Magnetic graphs for cavity quantum electrodynamics
Source: Sci Adv. 2026 Sep 25;12(39):eaee5566. doi: 10.1126/sciadv.aee5566 (PMC13614390; doi:10.1126/sciadv.aee5566)
Supplement: Supplementary file 1 [file sciadv.aee5566_sm.pdf]

Science Advances
AAAS

Supplementary Materials for

Magnetic graphs for cavity quantum electrodynamics

Sunkyu Yu *et al*.

Corresponding author: Sunkyu Yu, sunkyu.yu@snu.ac.kr; Xianji Piao, piao@uos.ac.kr; Namkyoo Park, nkpark@snu.ac.kr

Sci. Adv. **12**, eaee5566 (2026)
DOI: 10.1126/sciadv.aee5566

The PDF file includes:

Supplementary Notes S1 and S2
Figs. S1 and S2
Legend for code S1

Other Supplementary Material for this manuscript includes the following:

Code S1

Supplementary Text

Note S1. Distribution of v_1

Because stronger η enhances higher-order coupling as shown in Fig. 2 of the main text, it is easier to experience boundary effects even with a large cutoff $|n = N_{\max} = 200\rangle$. To guarantee the negligible boundary effects in analysing the FR graph through the graph Laplacian eigenvalue λ_1, it corresponding eigenstate v_1 must have negligible weight near the boundary. Figure S1 demonstrates that the range of $\eta \leq 2.66$ satisfies this condition.

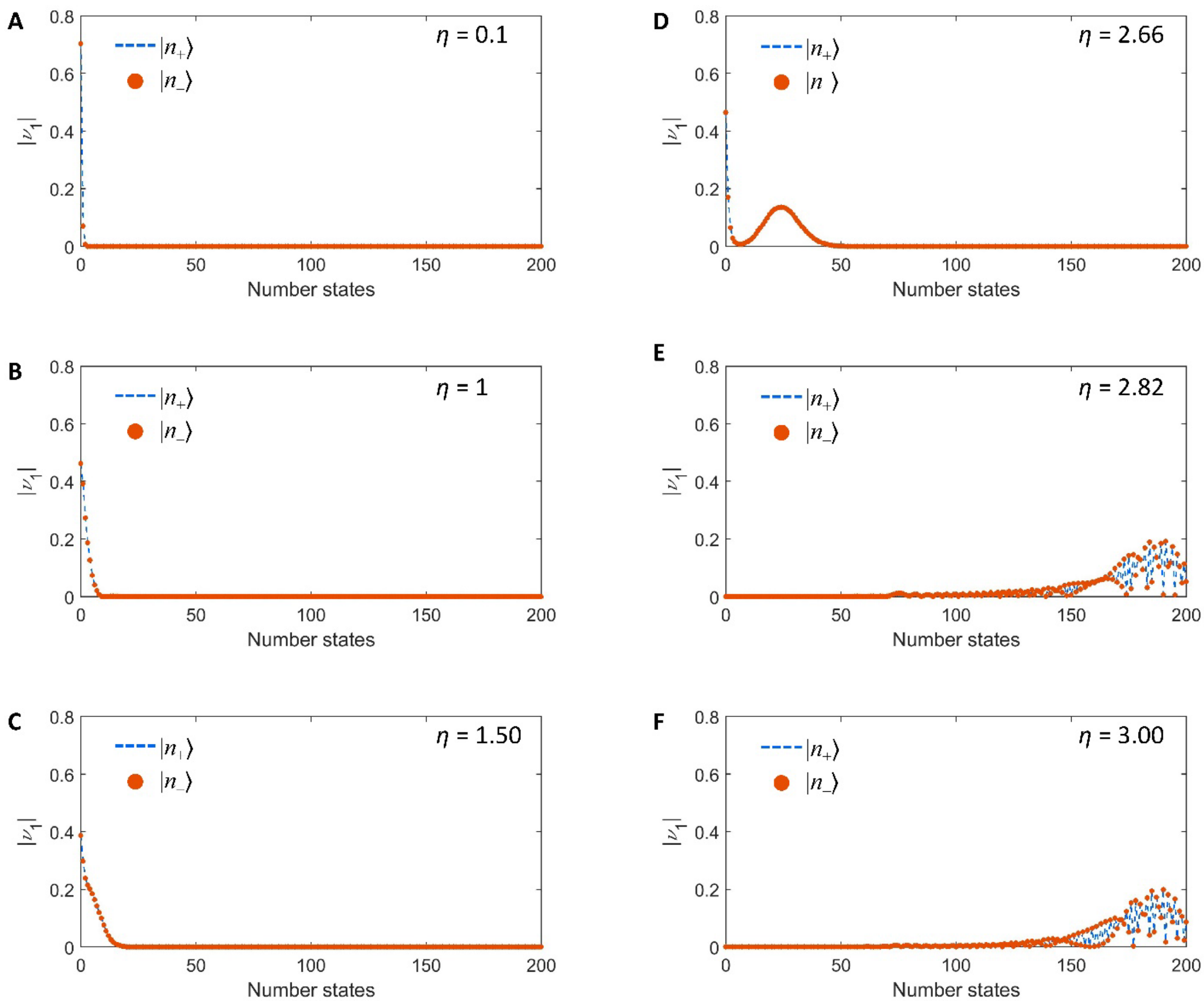

Fig. S1. Distributions of the Laplacian eigenvector v_1. η-dependent distributions of $|v_1|$ over the atomic and number states $|n_\pm\rangle$: **(A)** $\eta = 0.1$, **(B)** $\eta = 1$, **(C)** $\eta = 1.50$, **(D)** $\eta = 2.66$, **(E)** $\eta = 2.82$, and **(F)** $\eta = 3.00$.

Note S2. Loop formation in the FR graph

Figure S2A illustrates loop formation in the FR graph, where p_{Connect} denotes the probability of forming connected loops across 2×10^4 random subgraph realizations. When a loop is formed, it can exhibit either a trivial or nontrivial hopping phase. The probabilities of these two cases over all realizations are denoted as $p_{\mathrm{Nontrivial}}$ and p_{trivial}, (Fig. S2B), satisfying $p_{\mathrm{Connect}} = p_{\mathrm{Nontrivial}} + p_{\mathrm{trivial}}$.

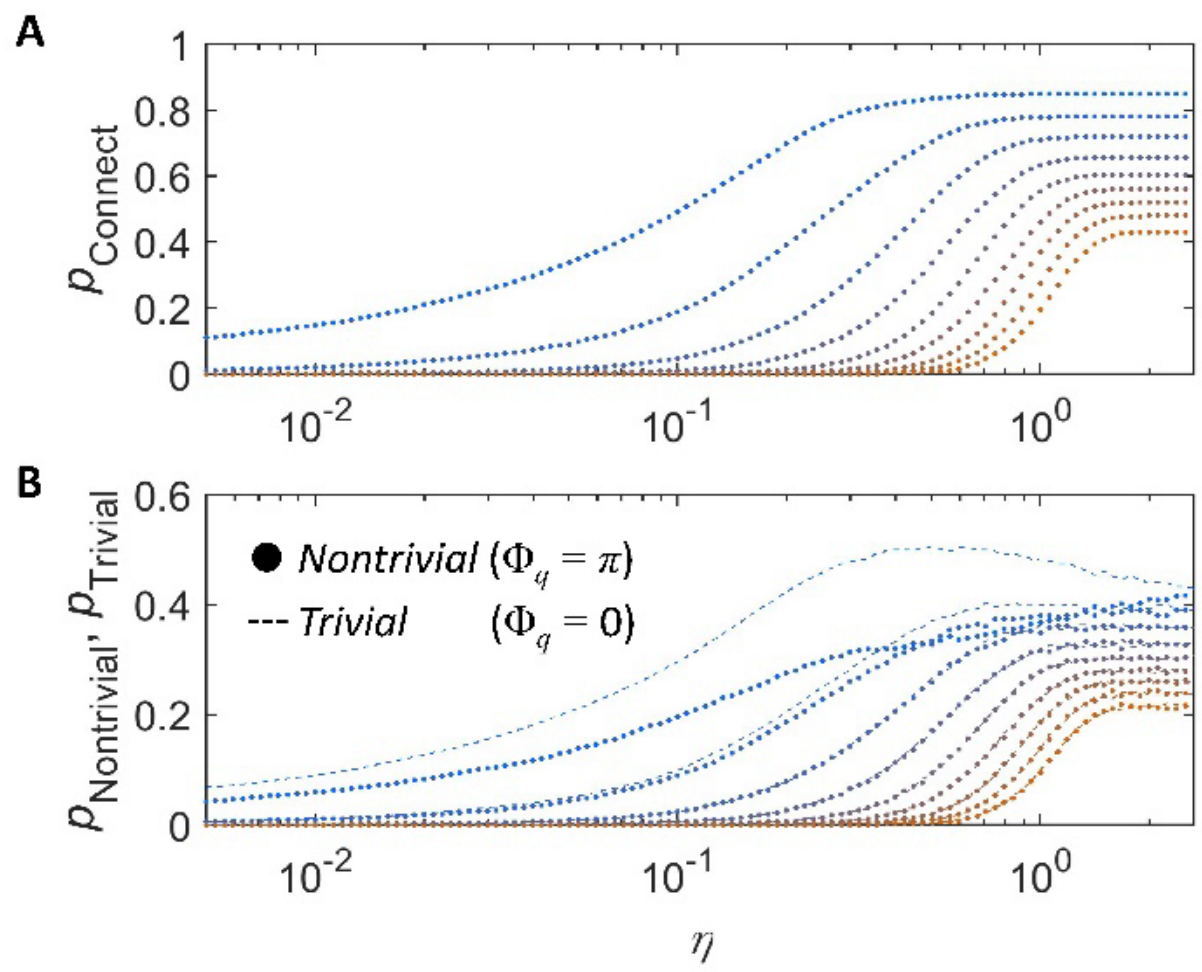

Fig. S2. Probabilities of loop formation. Loop formation probabilities as functions of η: (**A**) p_{Connect}, and (**B**) $p_{\mathrm{Nontrivial}}$ and p_{Trivial}. All the other parameters are the same as those in Fig. 4 in the main text.

Code S1. Source code for the Apollonian butterfly energy spectra.
This archive contains the MATLAB (R2023b) source code, ‘Q-ROS’. All data and figures presented in the manuscript can be fully reproduced by executing the scripts prefixed with ‘R003_’. Detailed instructions, along with descriptions of the core functionalities for the entire codes, are available in the included ‘README.txt’ and ‘Manual_QME_Ver_01_00_250612.pdf’ files.
